# HMGB1 knockdown increases MM cell vulnerability by regulating autophagy and DNA damage repair

**DOI:** 10.1186/s13046-018-0883-3

**Published:** 2018-08-29

**Authors:** Xing Guo, Donghua He, Enfan Zhang, Jing Chen, Qingxiao Chen, Yi Li, Li Yang, Yang Yang, Yi Zhao, Gang Wang, Jingsong He, Zhen Cai

**Affiliations:** 1 Bone Marrow Transplantation Center, The First Affiliated Hospital, College of Medicine, Zhejiang University, Hangzhou, 310003 Zhejiang China; 2 Quzhou People’s Hospital, Quzhou, 324000 Zhejiang China

**Keywords:** HMGB1, DNA damage, Multiple myeloma, Autophagy

## Abstract

**Background:**

With the development of novel therapeutic agents, the survival of multiple myeloma (MM) patients has much improved. However, the disease is incurable due to drug resistance. Previous studies have found that high-mobility group box 1 (HMGB1) is involved in inflammation, angiogenesis, DNA damage repair, and cancer invasion, progression, metastasis and drug resistance and that high HMGB1 expression is associated with poor MM prognosis, yet the role and mechanism of HMGB1 in MM remains unclear.

**Methods:**

Through gene expression and Oncomine database analyses, we found that HMGB1 is associated with a poor prognosis in MM patients. RNA interference together with gene array analysis, cell proliferation and apoptosis assays, autophagy detection assays, western blotting, and in vivo xenograft models were employed to evaluate the effect of HMGB1 and the mechanism involved in MM drug resistance.

**Results:**

MM cell lines and primary MM samples were found to express high levels of HMGB1, which was negatively associated with the 3-year survival of MM patients. HMGB1 knockdown in MM cells enhanced the inhibitory effect of chemotherapy with dexamethasone (Dex) via apoptosis induction. Furthermore, downregulation of HMGB1 activated the mTOR pathway, inhibited autophagy and increased DNA damage induced by Dex by modulating expression of related genes. In vivo, xenograft models showed that after Dex treatment, the tumor burden of HMGB1-knockdown mice was decreased compared with that of control mice.

**Conclusions:**

Our research shows that HMGB1 participates in autophagy and DNA damage repair and that downregulation of HMGB1 enhances the sensitivity of MM cells to Dex, suggesting that HMGB1 may serve as a target for MM treatment.

## Background

Multiple myeloma (MM) is one of the most commonly diagnosed hematological malignancies, second only to lymphoma. Increased blood calcium levels, renal insufficiency, anemia, and bone lesions (CRAB) are the major clinical features of MM [1]. Although the use of novel therapeutic agents (e.g., bortezomib, carfilzomib, thalidomide, lenalidomide) and autologous stem cell transplantation have significantly improved the survival of MM patients [2], most MM patients eventually relapse because of drug resistance, and MM remains incurable [3]. Hence, novel therapeutic targets and agents need to be developed for these patients.

High-mobility group box 1 (HMGB1), which is highly conserved, was first identified as a chromatin-associated protein [4]. HMGB1 binds to DNA to stabilize the nucleosome and plays an important role in DNA arrangement, replication, damage repair and transcription [5]. Studies have found that HMGB1 is involved in inflammation and angiogenesis as well as in the invasion, progression, metastasis, and drug resistance of cancers [6, 7]. HMGB1 mediates inflammation in cancer by upregulating pro-inflammatory cytokines (such as TNF, IL-1, and IL-6) and promoting carcinogenesis [8, 9]. Additionally, HMGB1 promotes expression of anti-apoptotic Bcl-2 family members and calnexin and inhibits pro-apoptotic cIAP expression, thus protecting cancer cells from apoptosis [10, 11]. Some researchers have found that HMGB1 is overexpressed in many hematological malignancies, such as leukemia and lymphoma, and that it mediates drug resistance through autophagy [12, 13]. The aim of this study was to explore the role of HMGB1 in MM cell proliferation and drug resistance.

## Methods

### Human MM cell lines and primary MM cells

Human RPMI8226, CAG and MM.1S MM cell lines were kindly provided by Dr. Qing Yi (Department of Cancer Biology, Lerner Research Institute, Cleveland Clinic, Cleveland, OH, USA) and cultured in RPMI 1640 medium (Thermo Scientific, HyClone, MA, USA) supplemented with 10% fetal bovine serum (Thermo Fisher Scientific, Gibco, MA, USA) and 1% L-glutamine at 37 °C in a humidified atmosphere with 5% CO_2_. Bone marrow tissues and primary CD138+ cells from the bone marrow of MM patients were obtained after receiving informed consent from the donors and approval from the Ethics Committee of First Affiliated Hospital, College of Medicine, Zhejiang University. CD138+ cells were by positive selection collected using CD138 microbeads (Miltenyi Biotech, CA, USA).

### Reagents and antibodies

Dexamethasone and dimethyl sulfoxide (DMSO) were purchased from Sigma (St. Louis, MO, USA). Chloroquine (CQ, an autophagy inhibitor) and AZD6738 (an ATR inhibitor) were purchased from Selleck Chemicals (Houston, TX, USA). Annexin V Apoptosis Detection Kit APC/PI was obtained from BD Biosciences (NJ, USA). The following primary antibodies were used: anti-HMGB1 (Cell Signaling Technology (CST), #6893), anti-caspase-3 (CST, #6893), anti-Bcl-2 (CST, #6893), anti-Bcl-xl (CST, #6893), anti-PARP (CST, #6893), anti-DEPTOR (CST, #6893), anti-mTOR (CST, #6893), anti-p-mTOR (ser2448) (CST, #6893), anti-p70S6K (CST, #6893), anti-p-p70S6K (Thr389) (CST, #6893), anti-Akt (CST, #6893), anti-p-AKT (ser473) (CST, #6893), anti-LC3A/B (CST, #6893), anti-γH2A.X (CST, #9718), anti-Rad51 (Abcam, ab133534), Anti-β-actin (Sigma-Aldrich, A1978) and anti-GAPDH (ProteinTech, No. 60004–1).

### RNA interference

A lentivirus containing green fluorescence protein(GFP) with a short hairpin RNA (shRNA) against human HMGB1 and a control lentivirus were obtained from GenePharma (Shanghai, China), transfected into MM cells according to the manufacturer’s instructions, and screened with puromycin. The interference sequence against HMGB1 was 5’-GGACAAGGCCCGTTATGAA-3′, and the control sequence was 5’-TTCTCCGAACGTGTACGT-3′.

### Semi-quantitative real time-polymerase chain reaction (qRT-PCR)

Total RNA was extracted from cells and reversed transcribed with a Prime Script RT reagent kit from TaKaRa (Otsu, Japan) according to the manufacturer’s instructions. The expression levels of target genes were analyzed by qPCR using a SYBR® Premix Ex Taq™ II Kit from Bio-Rad (CA, USA). Expression of the housekeeping gene GAPDH was used as an internal control. The following primers were synthesized by Tsingke (Beijing, China) (primer sequence 5′-3′): forward GCTCAGAGAGGTGGAAGACCA and reverse GGTGCATTGGGATCCTTGAA for human HMGB1; forward TTGGTATCGTGGAAGGACTCA and reverse TGTCATCATATTTGGCAGGTTT for human GAPDH; forward TTAGCAGACCGGGGCATTATT and reverse GAAGGTGCCGTCATCCTTTCT for human DEPTOR; forward GGTGGGGTCATGTGTGTGG and reverse CGGTTCAGGTACTCAGTCATCC for human Bcl-2; and forward GAGCTGGTGGTTGACTTTCTC and reverse TCCATCTCCGATTCAGTCCCT for human Bcl-xl.

### Western blot analysis

Total protein from MM cells was extracted with radioimmunoprecipitation assay (RIPA) buffer, and supernatants were collected for western blot assays. Proteins (20–40 μg) were separated on 8–12% pre-made protein electrophoresis gels and transferred to polyvinylidene difluoride membranes (Merck Millipore, Germany). The membranes were blocked with 5% nonfat milk or bovine serum albumin (BSA) for 1–2 h and then incubated with specific primary antibodies overnight at 4 °C. The membranes were washed for 15 min in Tris-buffered saline with Tween 20 (TBS-T) four times and then incubated with secondary antibodies at room temperature for 1 h. The membranes were washed with TBS-T, and bands were detected using an exposure meter (Bio-Rad, CA, USA) with an enhanced chemiluminescence detection kit for HRP (Biological Industries, Israel, Beit Haemek Ltd.).

### Cell proliferation assays

Cell Counting Kit-8 (CCK8) from Dojindo (Japan) was used to evaluate MM cell proliferation. MM cells (1–2 × 10^5^ cells/ml) were plated in 24-well plates and then treated or not with Dex at 37 °C in a humidified atmosphere with 5% CO_2_. After 1–7 days, the cells were transferred to 96-wells plates (100 μL/well) and treated with 10 μL of CCK8 reagent. The 96-well plates were incubated at 37 °C for 1–2 h. Absorbance was measured at 490 nm using a microplate reader (Bio-Rad, Model 680), and the following formula was used to calculate cell viability: cell viability (%) = OD value of test sample/OD value of control sample × 100%.

### Flow cytometry: Apoptosis, cell cycle and autophagy detection

To detect apoptotic cells, a sample of 1 × 10^5^ MM cells/mL (RPMI8226, CAG, or MM.1S) was plated in 12-well plates and incubated with Dex with or without CQ or AZD6738 for 48 h. The cells were harvested, washed twice with phosphate-buffered saline (PBS), resuspended in 100 μL of staining buffer and stained with Annexin V–APC/PI according to the manufacturer’s instructions. The cells were detected using flow cytometry, and the data were analyzed using FlowJo 7.6.1.

RPMI8226 and CAG cells (2 × 10^5^ cells/well) were cultured with Dex (0, 50, or 100 μM) in six-well plates for 48 h, washed twice with PBS and permeabilized with precooled 75% ethanol at 4 °C overnight. The next day, the cells were washed twice with PBS and incubated with 500 μL of PI for 30 min at room temperature according to the manufacturer’s instructions. The cells were detected using flow cytometry (BD Biosciences, CA, USA), and the data were analyzed using ModFit software (version 3.2, Verity Software House).

RPMI8226 cells (1 × 10^5^ cells/ml) were treated with Dex (100 μM) in 12-well plates for 48 h. The cells were harvested, and autophagy was detected by flow cytometry with Enzo Cyto-ID Autophagy Detection Kit (Enzo Life Sciences, NY, USA) according to the manufacturer’s instructions.

### Affymetrix HTA 2.0 array

MM cells before and after HMGB1 knockdown were sent in TRIzol reagent to Biotechnology Corporation (Shanghai, China), and changes in gene expression were assessed according to the manufacturer’s protocol.

### Human tumor xenografts in NOD–SCID mice

To observe the role of HMGB1 in vivo, a xenograft model of human myeloma was established. Four-week-old male NOD–SCID mice were purchased from Vital River Laboratory Animal Technology Co., Ltd. (Beijing, China); 7 × 10^6^ RPMI8226 cells (transfected with control or HMGB1 shRNA virus) were subcutaneously injected into the right flank of the mice. After approximately 1 week, when the established tumors had reached approximately 100–130 mm^3^, the mice were randomly divided into four groups and intraperitoneally injected with either vehicle or Dex [15 mg/(kg/day) for 10 days]. The tumor diameter was measured using calipers on day 1, day 3, day 5, day 8 and day 10, and the tumor volume was calculated as 4π/3 × (a/2)^2^ × b/2, where a is the width and b is the length. All experiments followed the procedures and protocols of the Animal Ethical Committee of the First Affiliated Hospital, College of Medicine, Zhejiang University.

### Immunohistochemistry

Tumor tissue samples extracted from the NOD–SCID mice were fixed in 4% paraformaldehyde, and immunohistochemical staining was performed by Servicebio (Wuhan, China).

### Statistical analysis

All results are presented as means ± standard deviation (SD). A two-tailed Student’s t-test was used to determine significant differences between two groups. All *p* values less than 0.05 were considered statistically significant. All analyses were performed using GraphPad Prism 5.0 (GraphPad Software, CA, USA).

## Results

### HMGB1 was highly expressed in MM cells, and its expression was negatively associated with MM patient survival

**Fig. 1:**
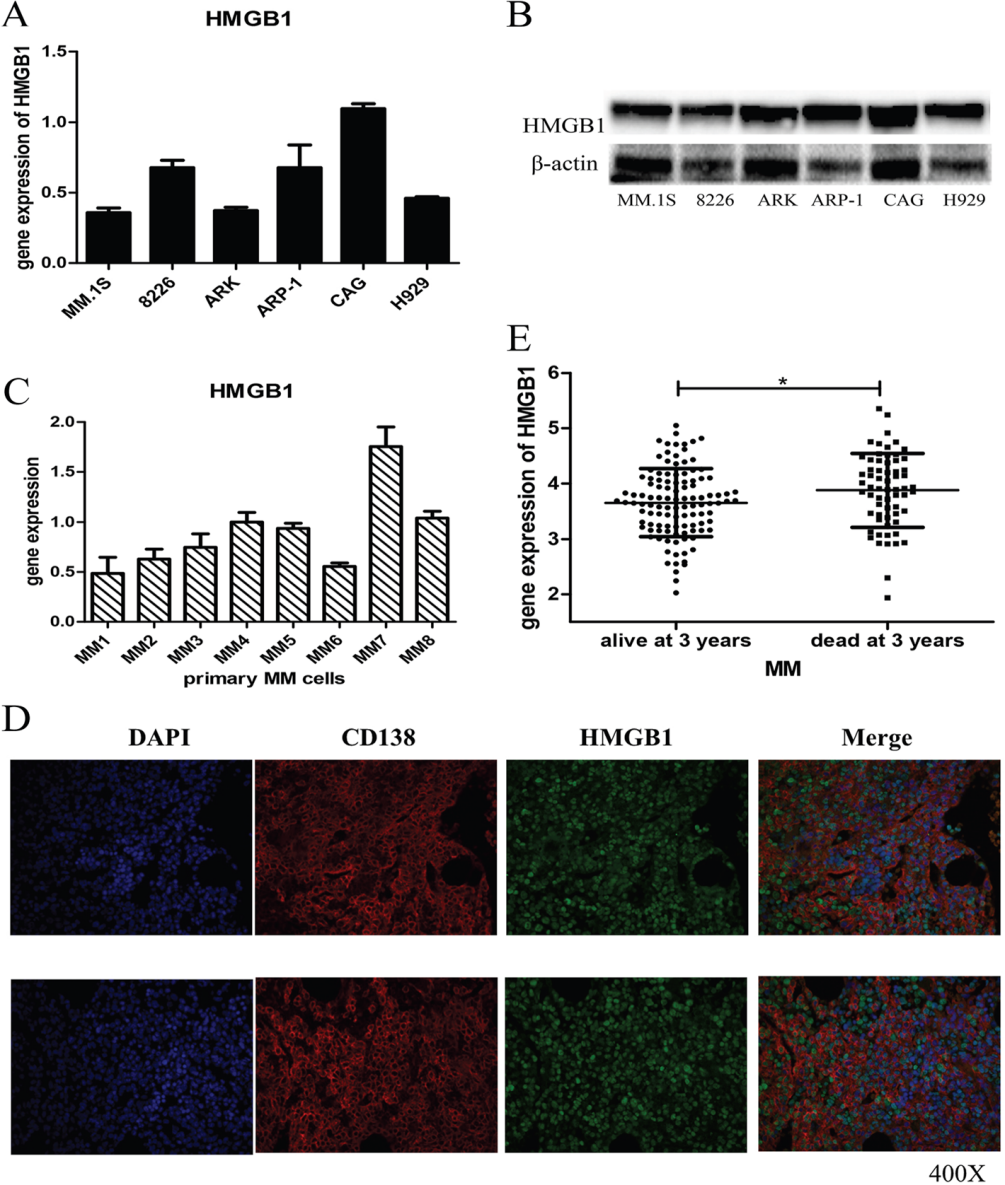
Expression of HMGB1 in MM cells. **a**, **b**: qRT-PCR (**a**) and western blotting (**b**) results of HMGB1 expression in MM cell lines; **c**: qRT-PCR results of HMGB1 expression in primary MM cells (cells from the bone marrow of MM patients sorted by CD138 antibody beads); **d**: Expression of HMGB1 in the bone marrow tissue of MM patients, as evaluated by immunofluorescence; **e**: HMGB1 expression was negatively associated with MM patient 3-year survival according to the analysis of the Oncomine database (*p < 0.05)

Expression of HMGB1 in MM cell lines and primary MM samples was detected. qRT-PCR and western blotting showed that the MM cell lines and primary MM samples assessed both expressed HMGB1, as shown in Fig. 1a-c. According to the immunofluorescence analysis of bone marrow tissues of MM patients presented in Fig. 1d, CD138+ plasma cells expressed HMGB1. Furthermore, data from Oncomine indicated that expression of HMGB1 was negatively associated with MM patients 3-year survival (Fig. 1e).

### HMGB1 knockdown enhanced the inhibitory effect of chemotherapy in MM cells

**Fig. 2:**
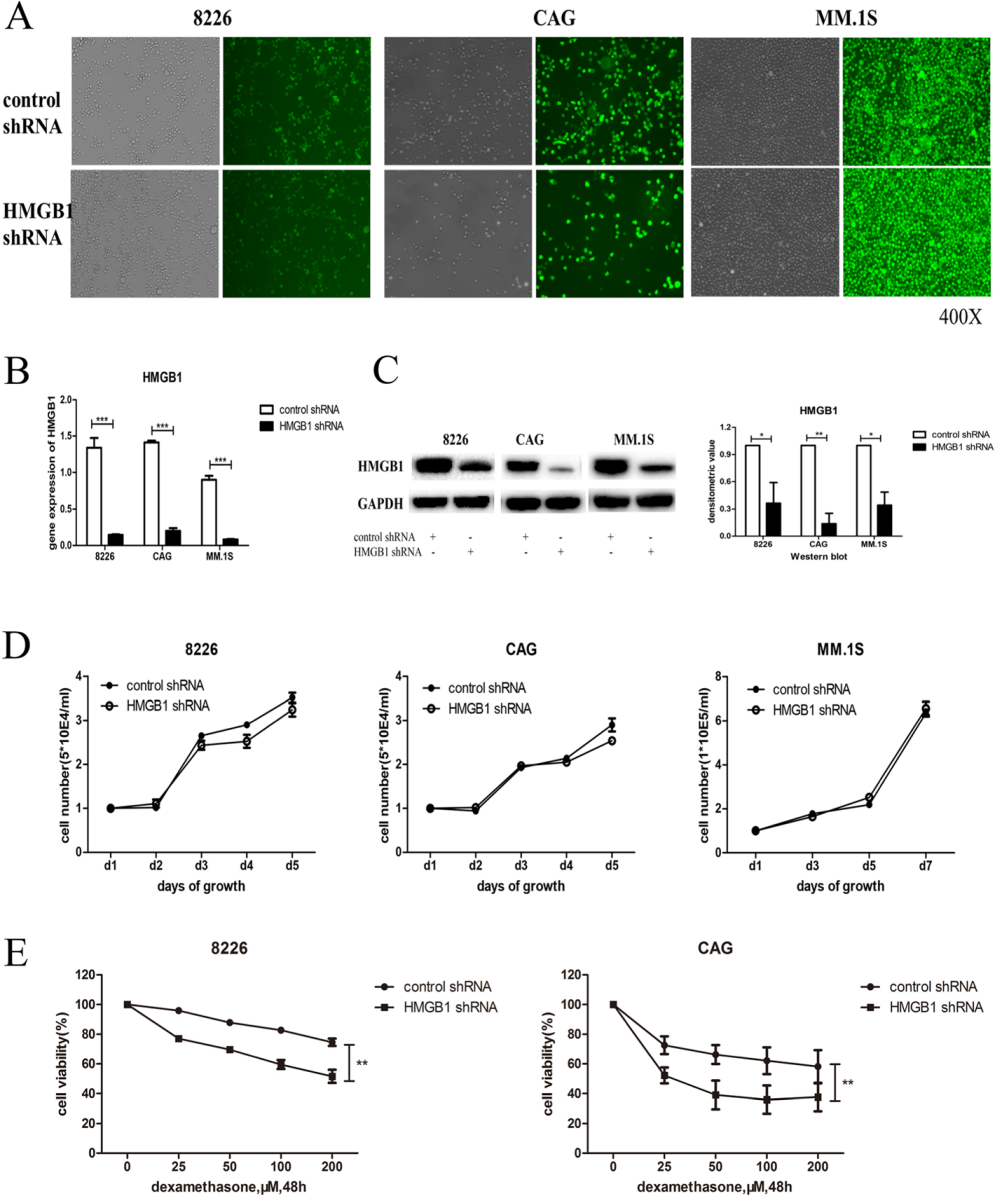
HMGB1 knockdown enhanced the inhibitory effect of chemotherapy in MM cells. **a**: MM cells (RPMI8226, CAG, MM.1S) were transfected with HMGB1 knockdown lentivirus, and the transfection effect was observed with a fluorescence microscope; **b**, **c**, **d**: qRT-PCR (**b**), western blot and densitometric analysis of the western blot results (**c**) were used to verify HMGB1 knockdown effect (*n* = 3); **d**: The CCK8 assay was used to determine the proliferation of MM cells after HMGB1 knockdown (n = 3, *p* > 0.05); **e**: The CCK8 assay was used to analyze the inhibitory effect of Dex after HMGB1 knockdown (*n* = 3, *P*>0.05) (*P<0.05,**P<0.01,***P<0.001)

To investigate the role of HMGB1 in the MM cell response to chemotherapy, MM cells were transfected with HMGB1-knockdown lentivirus, and a CCK8 assay was used to determine the proliferation of MM cells in the presence or absence of dexamethasone (Dex). As depicted in Fig. 2a-c, HMGB1 was significantly downregulated after lentiviral transfection. Although no difference in proliferation was observed between HMGB1-knockdown and control MM cells (*p* > 0.05; Fig. 2d), a significantly enhanced inhibitory effect of Dex was found in HMGB1-knockdown cells compared with wild-type HMGB1-control cells (*p* < 0.05; Fig. 2e).

### HMGB1 knockdown increased Dex-induced MM apoptosis

**Fig. 3:**
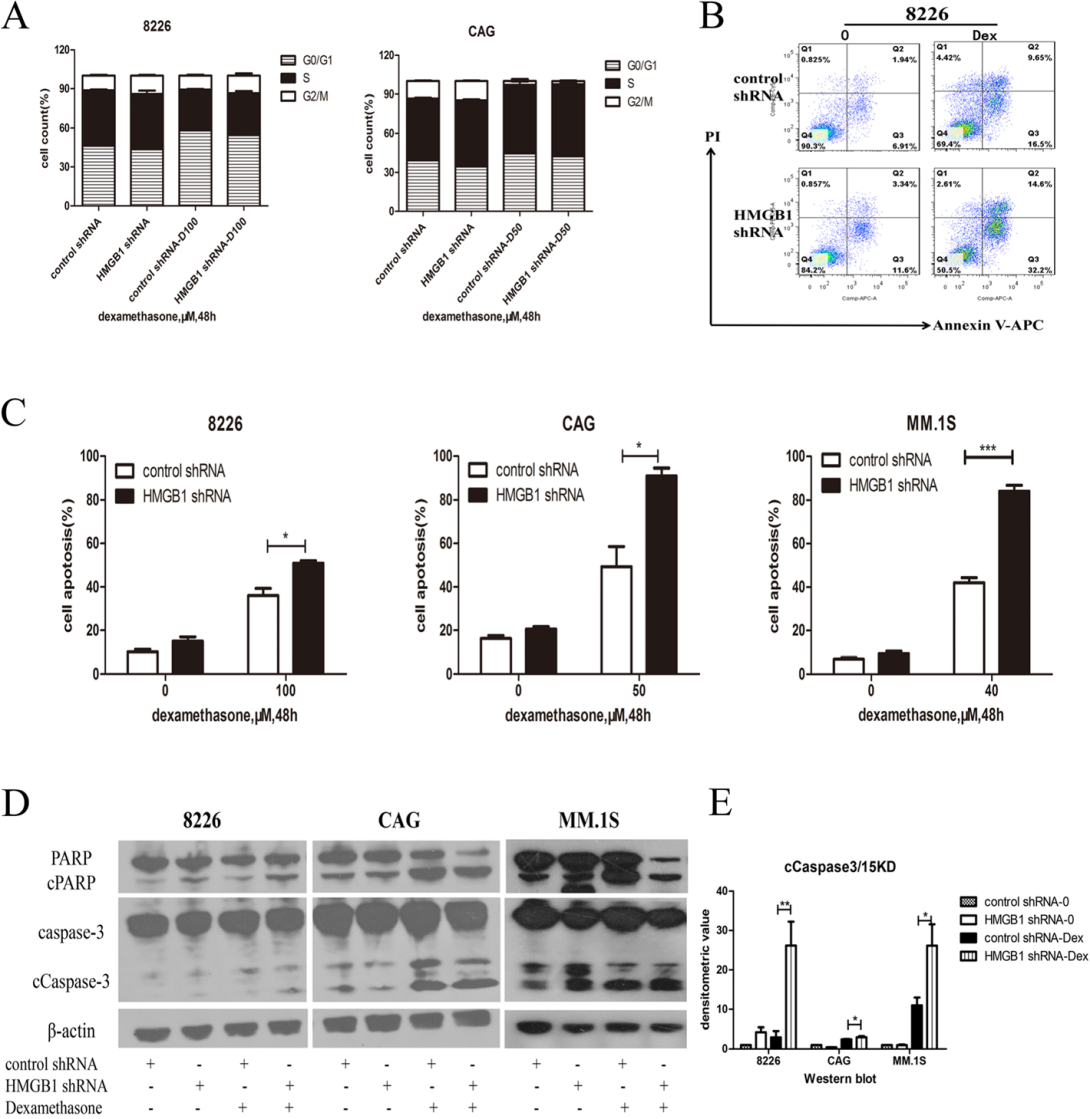
HMGB1 knockdown increased Dex-induced MM cell apoptosis. **a**: RPMI8226 and CAG were treated with Dex for 48 h, and cell cycle distribution was detected by flow cytometry (*n* = 3, *p* > 0.05); **b**: Representative flow cytometry results showing RPMI 82286 (transfected with control shRNA or HMGB1 shRNA) apoptosis after treatment with Dex (100 μM) for 48 h; **c**: Histograms showing the results of the analysis of cell apoptosis induced by Dex (*n* = 3); **d**: Western blotting was performed to detect the expression of c-caspase-3 and c-PARP induced by Dex after HMGB1 knockdown; **e**: Histograms showing the densitometric analysis of the 15 KD fragment of c-caspase-3 (*P<0.05,**P<0.01,***P<0.001)

Next, flow cytometry was performed to evaluate the cell cycle and apoptosis in MM cells. As illustrated in Fig. 3a, the results showed no differences in the cell cycle among the two groups. However, Dex-induced apoptosis of MM cells was significantly increased when HMGB1 expression was suppressed (*p* < 0.05; Fig. 3b-c). Moreover, results of western blotting revealed increased levels of Dex-induced cleaved (c)-caspase-3 and cleaved poly(ADP-ribose) polymerase (c-PARP) after HMGB1 knockdown, as shown in Fig. 3d-e.

### Affymetrix HTA 2.0 array

**Fig. 4:**
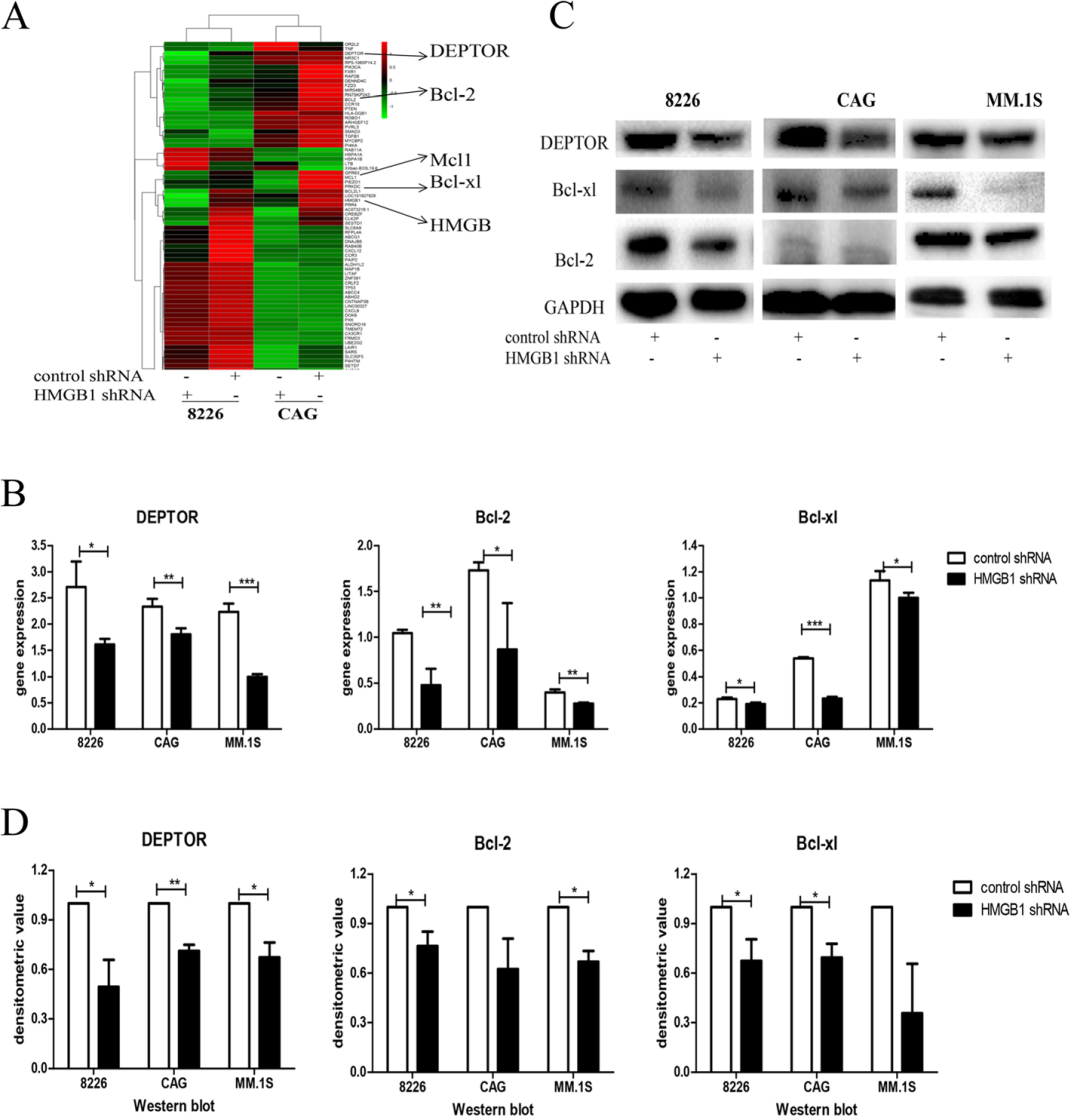
Changes of MM survival and apoptosis related genes after HMGB1 knockdown. **a**: Heatmap of the Affymetrix HTA 2.0 array results in RPMI8226 and CAG cells showing that the DEPTOR, Bcl-2, Bcl-xl, and Mcl1 levels were decreased; **b**, **c**,: qRT-PCR (B) and western blotting (**c**) were used to verify the array results; **d**: Densitometric analysis of the protein levels of DEPTOR, Bcl-2, and Bcl-xl (*n* = 3, *p < 0.05, **p < 0.01, ***p < 0.001)

To determine the mechanism by which HMGB1 knockdown enhanced the inhibitory effect of Dex, an Affymetrix HTA 2.0 array was employed to compare changes in gene expression levels between HMGB1 knockdown and control cells. As depicted in Fig. 4a, the levels of MM survival- and apoptosis-related genes such as DEPTOR, Bcl-2, Bcl-xl, and Mcl1 were decreased. Verification of these results by qRT-PCR and western blotting demonstrated that both anti-apoptotic (Bcl-2, Bcl-xl) and MM survival-related (DEPTOR) genes were decreased in knockdown cells compared to control cells (Fig. 4b-d).

### The mTOR pathway was activated and autophagy was inhibited by HMGB1 knockdown

**Fig. 5:**
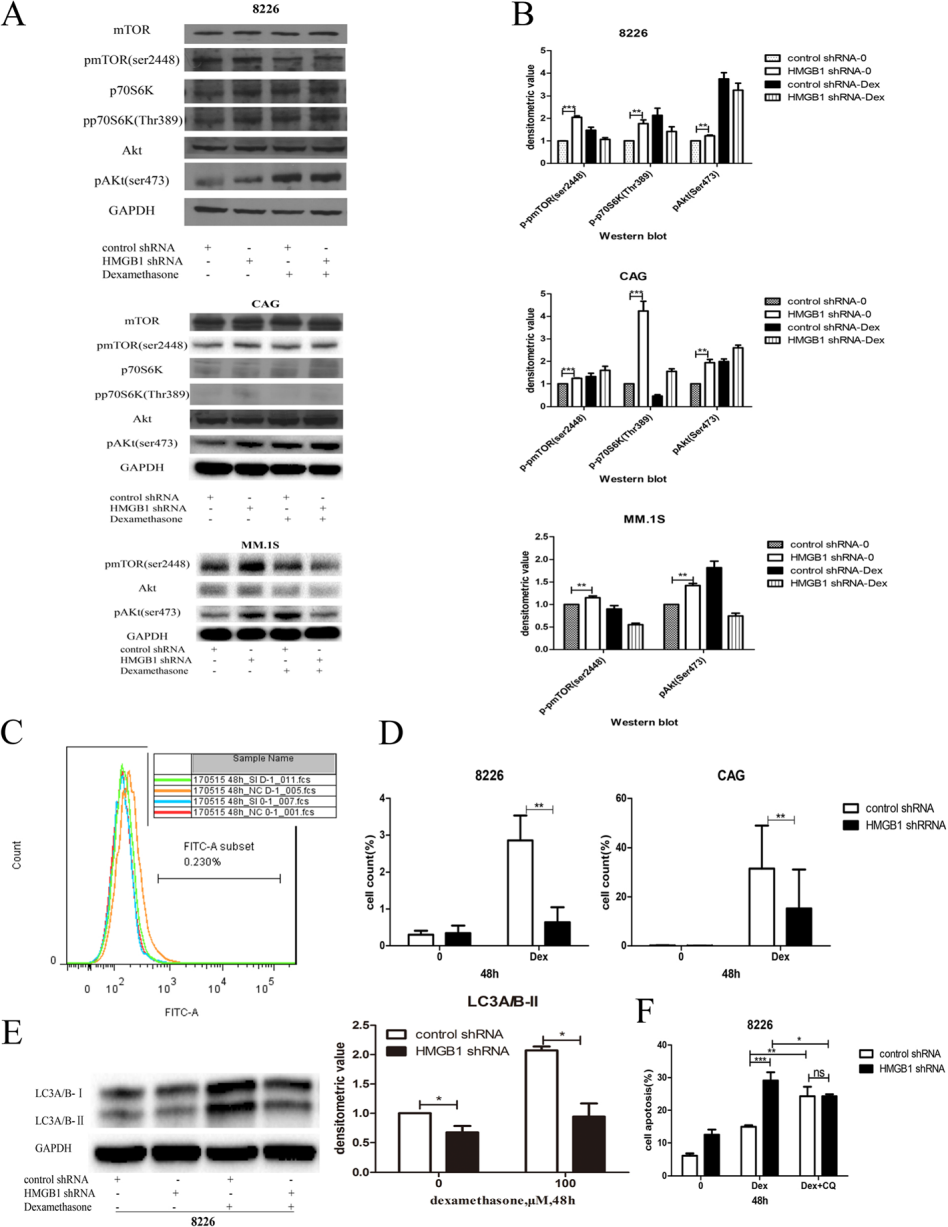
mTOR pathway was activated, autophagy was inhibited after HMGB1 knockdown and the enhanced sensitivity effect of HMGB1 knockdown was blocked by CQ (an autophagy inhibitor). **a**: Western blotting was performed to analyze changes in the mTOR pathway after HMGB1 knockdown; the phosphorylation of p70S6K (substrate of the mTORC1 complex) and AKT-ser473 (substrate of the mTORC2 complex) were both increased; **b**: Densitometric analysis of the phosphorylation of mTOR, p70S6K and AKT; **c**: Representative results showing the fluorescence intensities in RPMI8226 after Dex (100 μM) exposure using the Cyto-ID Autophagy Detection Kit with flow cytometry, control shRNA without Dex (red line), HMGB1 shRNA without Dex (blue line), control shRNA with Dex (orange line), and HMGB1 shRNA with Dex (green line); **d**: Histograms showing the analysis results of the MM cell lines(RPMI8226, CAG) autophagy fluorescence intensities induced by Dex after HMGB1 knockdow by Cyto-ID Autophagy Detection Kit n (*n* = 3); **e**: Western blotting was performed to detect the Dex-induced expression of LC3A/B-I/II after HMGB1 knockdown and the histograms showing the densitometric analysis of LC3A/B-II; **f**: Histograms showing the analysis results of RPMI8226 (transfection with control shRNA or HMGB1 shRNA) apoptosis induced by Dex (100 μM) in the presence of CQ (1 μM, an autophagy inhibitor) (*P<0.05,**P<0.01,***P<0.001)

According to the results of the Affymetrix HTA 2.0 array, DEPTOR was downregulated after HMGB1 knockdown. As previous studies have shown that DEPTOR plays an important role in the mTOR pathway and autophagy [14, 15], western blotting was performed to analyze changes in signaling pathways after HMGB1 knockdown. As shown in Fig. 5a-b, phosphorylation of both p70S6K (a substrate of the mTORC1 complex) and AKT-ser473 (a substrate of the mTORC2 complex) was increased in HMGB1-knockdown MM cells compared to control cells, suggesting that both pathways were activated after HMGB1 knockdown. A Cyto-ID Autophagy Detection Kit was then used with flow cytometry to examine the degree of autophagy induced in MM cells after HMGB1 knockdown. The results showed that after Dex exposure, a significant shift in the fluorescence peak to a higher intensity occurred in the RPMI8226 HMGB1 knockdown group (Fig. 5c-d). Additionally, western blot analysis revealed a significant decrease in the level of LC3A/B-II in the HMGB1 knockdown group treated with Dex, which suggested that the autophagy induced by Dex was inhibited after HMGB1 knockdown (Fig. 5e). To explore the role of autophagy in MM drug resistance, we detected apoptosis in RPMI8226 cells induced by Dex in the presence of CQ, an autophagy inhibitor. Flow cytometry analysis showed that Dex-induced apoptosis in RPMI8226 cells was significantly increased in the presence of CQ, both in control shRNA and HMGB1 knockdown groups. In contrast, no significant of Dex-induced apoptosis between these two groups occurred in the presence of CQ, as shown in Fig. 5f, indicating that HMGB1 may induce MM cell drug resistance through autophagy.

### HMGB1 knockdown increased DNA damage induced by Dex

**Fig. 6:**
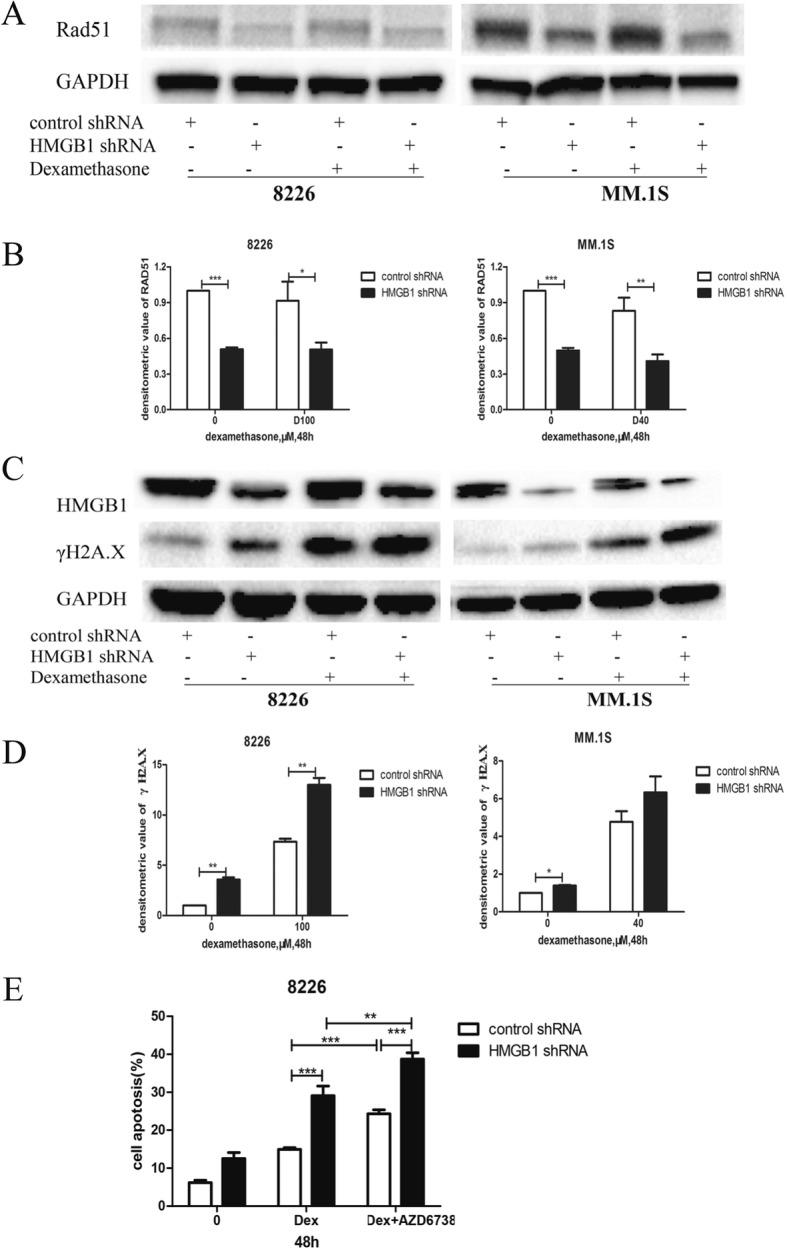
DNA damage induced by Dex was increased after HMGB1 knockdown. **a**, **c**: Western blotting was performed to evaluate the expression of the DNA damage and repair related proteins Rad51 (**a**) and γH2A.X (**c**), respectively, after HMGB1 knockdown with or without Dex exposure for 48 h; **b**, **d**:Densitometric analysis of Rad51 (**b**) and γH2A.X (**d**); **e**: Histograms showing the analysis results of RPMI8226 (transfection with control shRNA or HMGB1 shRNA) apoptosis induced by Dex (100 μM) in presence of AZD6738 (2 μM, an ATR inhibitor) (*P<0.05,**P<0.01,***P<0.001)

As HMGB1 is involved in DNA damage repair [16], γH2A.X, an important DNA damage marker [17], and Rad51, a DNA damage repair protein [18], were detected by western blotting. As presented in Fig. 6a-d, Rad51 expression was significantly decreased and that of γH2A.X increased with or without Dex treatment when HMGB1 was silenced. Furthermore, we detected apoptosis in RPMI8226 cells induced by Dex in the presence of AZD6738, an ATR inhibitor of the DNA damage repair pathway. According to flow cytometry analysis, apoptosis in these cells induced by Dex in the presence of AZD6738 was significantly increased in both the control shRNA group and the HMGB1 knockdown group, though the pro-apoptotic effect of HMGB1 knockdown was not blocked by AZD6738 (Fig. 6e).

### HMGB1 knockdown enhanced the sensitivity of MM cells to chemotherapy in vivo

**Fig. 7:**
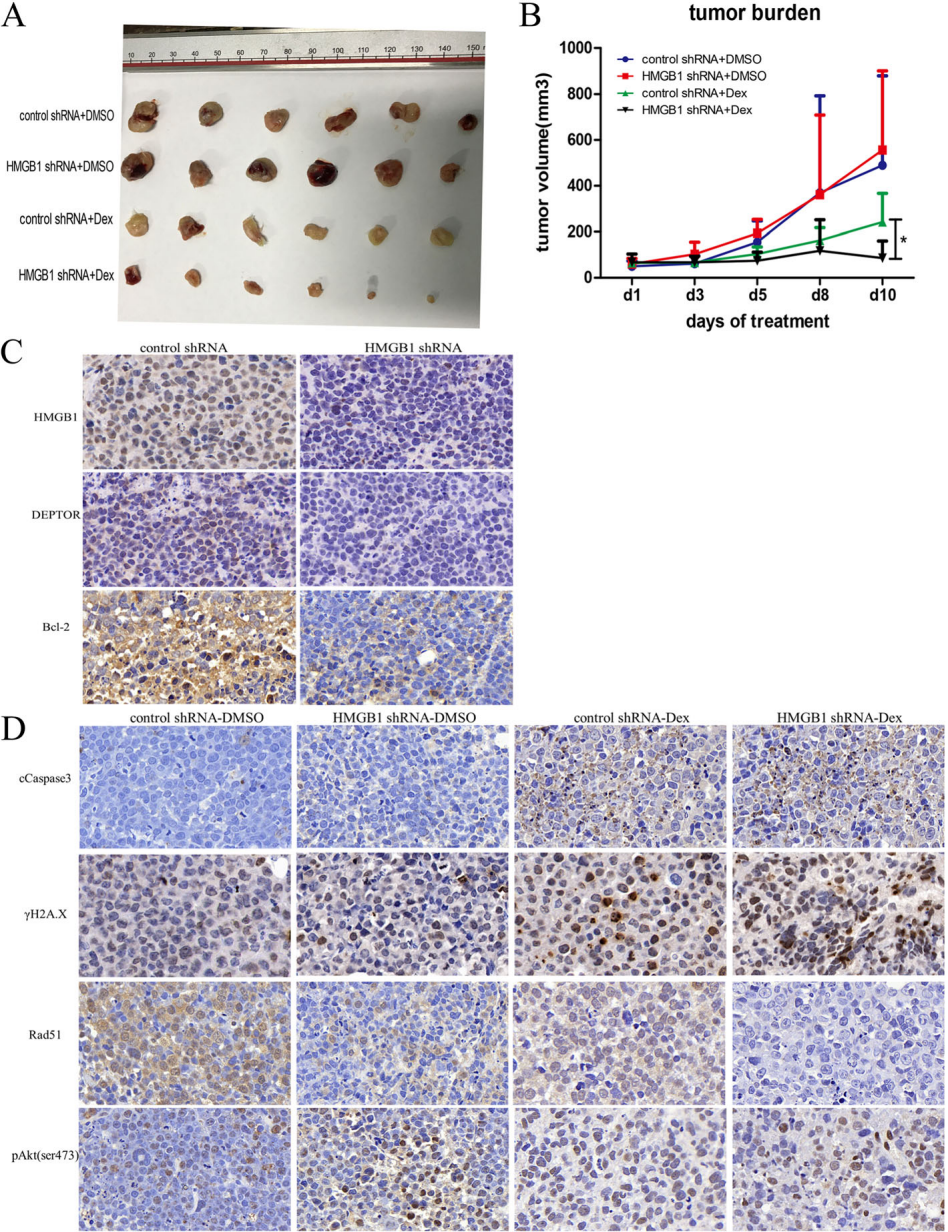
HMGB1 knockdown increased the sensitivity of MM cells to Dex in vivo. **a**, **b**: Tumor volume, as measured by Vernier calipers in the four different groups (six mice per group). **c**, **d**: Immunohistochemical analysis was performed to detect the expression of HMGB1, DEPTOR, Bcl-2, c-caspase-3,γH2A.X, Rad51 and pAKt(ser473)(magnification: 400×) (*P<0.05)

To evaluate the role of HMGB1 in MM cell proliferation and drug resistance in vivo, NOD–SCID MM mouse models using RPMI8226 cells (transfected with control or HMGB1 shRNA virus) were established and then treated with either Dex or an equal volume of DMSO as the control. As illustrated in Fig. 7a-b, after treatment with Dex, the tumor burden was lower in HMGB1 knockdown mice than in control mice (*p* < 0.05). Furthermore, the results of immunohistochemical analysis shown in Fig. 7c indicate that the expression levels of DEPTOR and Bcl-2 were decreased in the HMGB1 knockdown group. In addition, after treatment with Dex, c-caspase-3 and γH2A.X were significantly increased in the HMGB1 knockdown group compared with the control group (Fig. 7d), which was consistent with the in vitro results.

## Discussion

Using GEP analysis, a study by Roy in 2016 found HMGB1 to be overexpressed in the bone marrow plasma cells of MM patients compared with its expression in healthy donors, and the level of expression was also associated with survival in these patients. Our study showed that both MM cell lines and primary MM samples express HMGB1. Furthermore, using the Oncomine database, we found that expression of HMGB1 is negatively related with MM patient survival at 3 years, which suggests that HMGB1 plays a role in the development and drug resistance of MM.

Previous studies have demonstrated that downregulating HMGB1 in cancer cells such as gastric cancer [19], bladder cancer [20], and non-small cell lung cancer [21] through RNA interference (RNAi) or micro-RNA overexpression can inhibit cell proliferation and induce cell cycle arrest or apoptosis to increase the sensitivity of tumor cells to chemotherapy [5]. Another study in rectal cancer [22] found that HMGB1 knockdown by RNAi activated caspase-3 and PARP, downregulated Bcl-2 expression and eventually induced apoptosis in rectal cancer cells. In the present study, the expression levels of anti-apoptotic proteins Bcl-2 and Bcl-xl were downregulated after HMGB1 knockdown, indicating that suppressing HMGB1 expression can induce MM apoptosis by regulating Bcl-2 and Bcl-xl expression. Nonetheless, further investigation of the mechanism by which HMGB1 regulates protein expression is needed.

HMGB1 knockdown in chronic myeloid leukemia (CML) cells has a synergistic effect by inhibiting proliferation and inducing apoptosis [23]. Studies in leukemia cells have demonstrated that HMGB1 overexpression decreased leukemia cell sensitivity to chemotherapy (i.e., adriamycin, vincristine, cytarabine) and that downregulation of HMGB1 enhanced cell sensitivity to chemotherapy [12, 13]. Dex is a common traditional chemotherapy drug that is combined with novel agents for the treatment of MM [24–26]. Our study showed that interfering with HMGB1 expression increased the MM apoptosis induced by Dex, which suggests that HMGB1 knockdown also exerts a synergistic inhibitory effect with Dex in MM.

DEPTOR, which was first named by Timothy in 2009 as the product of the DEPDC6 gene, is an endogenous inhibitor of mTOR [27]. Expression of DEPTOR is low in many tumors, and previous studies have found that DEPTOR is overexpressed in MM cells with t(11;14) or t(14;16) translocation and that it may affect the drug response of MM cells. These studies have also reported that DEPTOR downregulation in MM cells activates mTORC1 and inhibits mTORC2, resulting in decreased phosphorylation of Akt-ser473, which in turn suppresses proliferation and promotes apoptosis in MM cells [14, 28]. But our gene expression array showed that DEPTOR was significantly decreased and that both the mTORC1 and mTORC2 pathways were activated after HMGB1 knockdown.

Research on autophagy in MM drug resistance is a hot topic [29, 30], and DEPTOR also induces MM drug resistance through autophagy [31–33]. Many previous studies have shown that endogenous HMGB1 can induce drug resistance through autophagy [34, 35]. For example, HMGB1 induces drug resistance by activating leukemia cell autophagy via the PI3K/Akt/mTORC1 pathway, and reduced expression of HMGB1 activates mTOR and promotes the phosphorylation of Akt and p70S6k, inhibiting leukemia cell autophagy and increasing drug sensitivity [12, 36]. Roy also found that lycorine inhibited autophagy in MM cells by downregulating HMGB1 expression [37]. In the present study, we showed that after HMGB1 knockdown, phosphorylation of Akt and p70S6k increased without Dex treatment and that basal autophagy decreased. We also found that the level of LC3A/B-II in RPMI8226 cells with HMGB1 knockdown was significantly decreased following Dex exposure, indicating that the autophagy process induced by Dex was reduced after HMGB1 knockdown. However, after Dex treatment, phosphorylation of p70S6K and Akt in the HMGB1-knockdown group was reduced compared to that in the HMGB1-control group, which was not correlated with inhibition of autophagy. Perhaps Dex also affects the mTOR pathway through HMGB1; thus, the activity of the mTOR pathway was not correlated with decreased autophagy after HMGB1 knockdown in the presence of Dex. Regardless, the effect of HMGB1 on Dex-induced mTOR pathway activation requires further study. We also found significantly increased levels of apoptosis induced by Dex in RPMI8226 cells in the presence of CQ, both in the control shRNA and HMGB1 knockdown groups; in the presence of CQ, there was no significant difference in Dex-induced cell apoptosis between the two groups, which suggested that HMGB1 may play a role in MM drug resistance by regulating autophagy through the DEPTOR/mTOR/Akt pathway.

Deregulation of DNA damage repair is one of the most important mechanisms in MM drug resistance [38, 39]. The Fanconi anemia/BRCA (FA/BRCA) DNA damage repair pathway can induce melphalan resistance in MM cells by promoting repair of interstrand DNA crosslinks (ICLs) and inhibiting the pathway enhances the response of MM cells to melphalan [40–42]. Previous studies have shown that HMGB1 can interact with various DNA repair proteins (e.g., RAG and XPA) to address DNA damage repair and to inhibit tumor cell apoptosis [16, 43]. In bladder cancer cells, the amount of DNA damage induced by radiotherapy was increased by more than twofold, that enhancing cell sensitivity to radiotherapy, when HMGB1 was knocked down [20]. Both internal and external factors can cause DNA damage, and there are many types of such damage, including single-strand breaks(SSBs) and double-strand breaks(DSBs) breaks. Shortly after the occurrence of a DSB, ATR recognizes the damage during DNA replication; H2A.X is then phosphorylated at serine 139 (γH2AX) by kinases of the PI3 family (including ATR and DNA-PKcs). Hence, γH2A.X is a key marker of DNA damage [17, 44]. Two main repair pathways are responsible for DSBs: non-homologous end joining (NHEJ) and homologous recombination (HR). Rad51 plays a major role in HR [18], and our results demonstrated that expression of Rad51 was decreased and that of γH2A.X increased when HMGB1 was knocked down, regardless of Dex treatment. As stated above, ATR is involved in DNA damage repair, and our study showed significantly increased levels of Dex-induced apoptosis in RPMI8226 cells in the presence of an ATR inhibitor (AZD6738) both in the HMGB1 knockdown group and the control group. Moreover, the pro-apoptotic effect of HMGB1 knockdown was not blocked by AZD6738, suggesting that HMGB1 knockdown may inhibit the repair of replication-dependent DNA damage, which is dependent on ATR, by activating components upstream of the kinase but not necessarily in an independent pathway. Nonetheless, further studies are needed to explore the specific mechanism.

Our in vivo experiments showed that after Dex treatment, the HMGB1-knockdown mice had a lower tumor burden than the control mice. Immunohistochemical analysis showed that the expression levels of DEPTOR, Bcl-xl and Rad51 were decreased and that the levels of c-caspase-3 and γH2A.X were significantly increased in the HMGB1 knockdown group, which was consistent with our in vitro results.

## Conclusions

Our study shows that HMGB1 may regulate expression of anti-apoptotic proteins and participate in the mTOR pathway and autophagy by regulating DEPTOR levels and may contribute to the repair of DNA damage induced by chemotherapy. The ultimate result is enhanced vulnerability of MM cells to Dex both in vitro and in vivo, suggesting that HMGB1 is a possible target for MM treatment.

## Abbreviations

CCK8: Cell Counting Kit-8
CML: Chronic myeloid leukemia
c-PARP: Cleaved poly(ADP-ribose) polymerase
CQ: Chloroquine
CST: Cell Signaling Technology
Dex: Dexamethasone
DMSO: Dimethyl sulfoxide
DSB: Double-strand break
FA/BRCA: Fanconi anemia/BRCA
HMGB1: High-mobility group box 1
HR: Homologous recombination
ICLs: Interstrand DNA crosslinks
MM: Multiple myeloma
NHEJ: Non-homologous end joining
RNAi: RNA interference
SSB: Single-strand break
